# Direct velocity measurements in high-temperature non-ideal vapor flows

**DOI:** 10.1007/s00348-021-03295-4

**Published:** 2021-09-10

**Authors:** Simone Gallarini, Fabio Cozzi, Andrea Spinelli, Alberto Guardone

**Affiliations:** 1 Energy Department, Politecnico di Milano, Milano, Italy via Lambruschini, 4A, 20156; 2 Department of Aerospace Science and Technology, Politecnico di Milano, Milano, Italy via La Masa, 34, 20156

## Abstract

Direct velocity measurements in a non-ideal expanding flow of a high temperature organic vapor were performed for the first time using the laser Doppler velocimetry technique. To this purpose, a novel seeding system for insemination of high-temperature vapors was specifically conceived, designed, and implemented. Comparisons with indirectly measured velocity, namely inferred from pressure and temperature measurements, are also provided. Nozzle flows of hexamethyldisiloxane (MM, C$_6$H$_{18}$OSi$_2$) at temperature up to $220\,^\circ \mathrm {C}$ and pressure up to 10 bar were taken as representative of non-ideal compressible-fluid flows. The relative high temperature, high pressure and the need of avoiding contamination pose strong constraints on the choice of both seeding system design and tracer particle, which is solid. A liquid suspension of tracer particles in hexamethyldisiloxane is injected through an atomizing nozzle in a high-temperature settling chamber ahead of the test section. The spray droplets evaporate, while the particles are entrained in the flow to be traced. Three different test cases are presented: a subsonic compressible nozzle flow with a large uniform region at Mach number 0.7, a high velocity gradient supersonic flow at Mach number 1.4 and a near-zero velocity gradient flow at Mach number 1.7. Temperature, pressure and direct velocity measurements are performed to characterize the flow. Measured velocity is compared with both computational fluid dynamics (CFD) calculations and velocity computed from pressure and temperature measurements. In both cases, the thermodynamic model applied was a state-of-the-art Helmoltz energy equation of state. A maximum velocity deviation of 6.6% was found for both CFD simulations and computed velocity.

**Graphical abstract:**

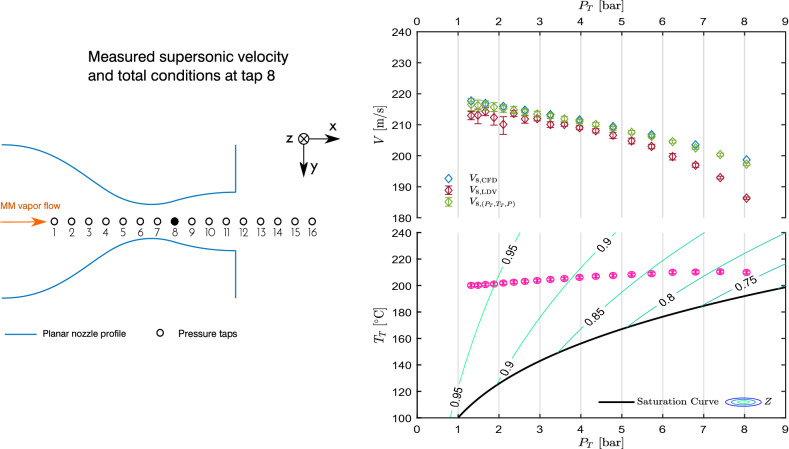

## Introduction

Compressible flows of fluids operating at thermodynamic conditions close to the vapor saturation line and the critical point are of interest in various applications in the oil and gas sector, in the chemical process industry, and in the energy field, including power systems as organic Rankine cycles (ORCs), supercritical carbon dioxide cycles (sCO$_2$), steam Rankine cycles and high temperature heat pumps. These are vapor or supercritical fluid flows, occurring at moderate to high temperature, including supercritical states, in components which are typically unconventional turbomachines, valves, heat exchangers, and pipelines. Such flows belong to the domain of non-ideal compressible fluid dynamics (NICFD), which also includes near-critical and two-phase fluid flows.

In the last two decades, considerable advancements were made in the thermodynamic modeling of fluids at highly non-ideal conditions, where non-ideal refers to deviation of the fluid behavior from the ideal gas law $Pv=RT$ (Span et al. 2001). Also, high-fidelity computational fluid dynamics (CFD) tools specifically conceived for highly non-ideal flows (Guardone and Vigevano 2002; Colonna and Rebay 2004; Cinnella and Congedo 2005; Vitale et al. 2015), and implementing state-of-the-art thermodynamic models (Span and Wagner 2003; van der Stelt et al. 2012; Thol et al. 2016), were developed and largely applied to perform fundamental studies and to design and analyze performances of the above described components.

Conversely, experiments required to verify theoretical findings and assess the accuracy of CFD tools are relatively recent, as a consequence of the hostile environment in which measurements have to be performed, which requires dedicated facilities to run the tests and specifically developed measurement techniques. Temperature measurements are obtained through thermocouples or resistance thermometers (depending on the required time resolution), while directional pressure probes provide, once calibrated, static and total pressure at the measuring point and the flow velocity via the fluid thermodynamic model, if the total temperature is measured. The stagnation pressure is of relevance if an evaluation of losses is required, as it is the case of components as turbomachines, valves, and pipelines. Differently from the perfect gas case, in non-ideal flows directional probes require a calibration procedure which is both fluid specific and thermodynamic condition specific. As an alternative, numerical calibration procedures can be adopted. Such techniques are under development, but not yet available if a high degree of accuracy is required. However, the pressure field can be measured for isentropic flows as the total pressure is conserved and the static pressure is measured through wall pressure taps. This is for instance the case of nozzle flows and uniform flows before/past oblique shocks and expansion fans, where the non-ideal pressure field was successfully measured by the authors in previous experimental campaigns (Spinelli et al. 2018; Zocca et al. 2019). As it is well known, the velocity field can be inferred from pressure and temperature field, by resorting to the thermodynamic model of the fluid. Indeed, since the velocity is obtained indirectly, its uncertainty is larger than the one of pressure and temperature. The uncertainty of the thermodynamic model should also be considered, even though it is negligible with respect to the one of operating conditions, if state-of-the-art thermodynamic models are adopted, see Congedo et al. (2011, 2013), Geraci et al. (2016) and Merle and Cinnella (2015).

In the field of non-ideal compressible flows, the above considerations raised the interest toward measurement methods which do not require calibration within reference flows, and specifically toward optical techniques such as schlieren imaging, laser Doppler velocimetry (LDV) and particle image velocimetry (PIV).

The schlieren technique (Spinelli et al. 2016, 2017; Zocca et al. 2018, 2019) is of relatively simple application in high-temperature non-ideal flows, but it is essentially qualitative, even though it can be exploited to measure the local Mach number, from Mach lines, and shock wave/expansion fan geometry (position, slope) (Cammi et al. 2021). However, this method can be used only in supersonic flows and with limited accuracy; moreover, to retrieve the velocity field from the Mach number one requires the application of the thermodynamic model, and thus the knowledge of at least two thermodynamic properties at the measuring point. Contrarily, both PIV and LDV techniques guarantee the direct measurement of the flow velocity, providing that the flow is properly inseminated, namely that the velocity slip between the fluid and the tracking particles is considerably small. Such velocity measurements are thus considered to be direct, oppositely to those inferred from temperature and pressure measurements via the fluid thermodynamic model, here referred to as indirect velocity measurements.

Direct velocity measurements complement the flow characterization performed through pressure and temperature measurements and are highly valuable in non-ideal flows, especially for components whose operation is strictly related to the velocity field, such as turbomachines, heat exchangers and pipelines. Moreover, a comparison between velocity either measured or inferred from pressure and temperature data via the thermodynamic model allows to assess the attainable accuracy in providing the velocity field of directional pressure probes, which are still under development for non-ideal flows. These reasons motivate the opportunity of measuring the velocity field though techniques which do not require fluid-specific and/or thermodynamic condition-specific calibration.

The flow seeding is particularly critical due to the need of introducing particles in a high temperature, high pressure and possibly condensing flow; also, fluid contamination must be avoided. Solid particles, chemically compatible and insoluble in the working fluid, are the only viable option, due to the high temperature they need to withstand to without significantly altering their dimension and density.

To the authors’ knowledge, no experimental studies about direct velocity measurements in high speed non-ideal flows were found in the literature up to date. A recent research (Valori et al. 2019) applied PIV to an unconventional vapor in highly non-ideal state. However, the analyzed conditions were low temperature and high pressure, and the study dealt with natural convection, thus the flow field featured extremely low velocity. Flows to be studied in the campaign presented here feature at the same time high temperature, high pressure and high velocity, thus making LDV (or PIV) implementation not straightforward.

Though a pointwise technique, LDV features, with respect to PIV, an easier implementation (the laser emitter and the receiver may be placed in the same device) and a higher attainable spatial and temporal resolution. For these reasons, LDV technique (extensively described in Albrecht et al. 2003) was selected in this work to obtain direct velocity measurements capable of providing, with temperature and pressure data, a complete and high spatial resolution characterization of high temperature non-ideal vapor flows.

In this work, experiments were carried out on isentropic, non-ideal expanding flows of hexamethyldisiloxane vapor (MM), which target operating conditions relevant to typical ORC turbine applications. The flow is characterized at selected locations along the axis of planar nozzles at different Mach number and thermodynamic conditions, from subsonic to supersonic regimes. Flow description is obtained by measuring total pressure and temperature, static pressure, and velocity, both directly measured through LDV and inferred from measured pressure and temperature via a suitable thermodynamic model.

Three different flows are analyzed: a subsonic flow at $M=0.7$, with almost zero velocity gradient, a supersonic flow at $M=1.7$, in a region of near-zero velocity gradient, and a supersonic flow at $M=1.4$, in a region of large velocity gradient. The experimental campaign was carried out, at the CREA Laboratory of Politecnico di Milano (Italy), on the Test Rig for Organic VApors (TROVA), a blow-down wind tunnel for organic vapors in non-ideal compressible flow conditions. The seeding particles selected for flow tracing are $0.2\,\upmu \mathrm {m}$ diameter titanium dioxide (TiO$_2$) particles, and a specifically conceived seeding system was designed and implemented. A liquid suspension of the tracer particle in the fluid to be tested is injected by an atomizer into the main flow at high temperature and pressure. The injection point is located in a plenum ahead of the test section. Due to the high temperature, the liquid fraction evaporates and solid particles trace the flow, allowing to measure the flow velocity.

At the measuring points, the directly measured velocity was compared to both velocity inferred from pressure and temperature measurements and to the one extracted from CFD calculation. This allowed to assess the consistency of different data sets and thus verifying the reliability of the implemented seeding system and the applicability of LDV in highly compressible and non-ideal vapor flows occurring at high temperature.

The paper is organized as follows. The experimental facility instrumentation and measurement techniques for pressure and temperature are described in Sect. 2. Section 3 presents experiments and nozzle design, while Sect. 4 discusses the choice of the tracer particle, the design of the seeding system and its operation. The employed LDV system is also presented. Section 5 describes velocity signals processing, while results are presented and discussed in Sect. 6. Finally, conclusions are drawn in Sect. 7.

## Experimental facility and techniques for pressure and temperature measurements

The experimental facility employed for this study is the Test Rig for Organic VApors, TROVA, a blow down wind tunnel designed to characterize non-ideal compressible flows of organic fluids of different nature; a detailed description of the facility can be found in Spinelli et al. (2013). To achieve the complete characterization of a fluid flow, the TROVA was conceived for the independent measurement of total pressure $P_T$, total temperature $T_T$, static pressure *P* and fluid velocity $V_f$.

Tests at a maximum pressure of ${50}\,{\mathrm{bar}}$ and maximum temperature of $400\,^{\circ }\mathrm{C}$ can be performed, providing that the working fluid does not undergo substantial decomposition at such temperature level. The fluid selected for the present campaign is siloxane MM, due to its widespread use in high-temperature ORC systems and the presence of a wide thermodynamic region where non-ideal effects are marked and thermal stability is preserved.

The TROVA implements a batch organic Rankine cycle, with a fixed test section replacing the turbine. The desired superheated or supercritical test conditions are reached by a isochoric heating of the fluid under scrutiny within a high pressure vessel, HPV. The tank pressure is higher than the test section stagnation pressure $P_{T}$, which can be regulated through a control valve and measured, along with total temperature $T_{T}$, in a plenum upstream of the test section. In all experimental runs, the test section is equipped with a planar nozzle. The fluid expands through the nozzle, where static pressure along the axis is measured; also, an optical access allows to perform LDV measurements and schlieren visualizations of the flow field. The exhausted vapor is discharged and condensed into a low pressure vessel, and liquid compression to the HPV through a metering pump closes the loop.

**Fig. 1:**
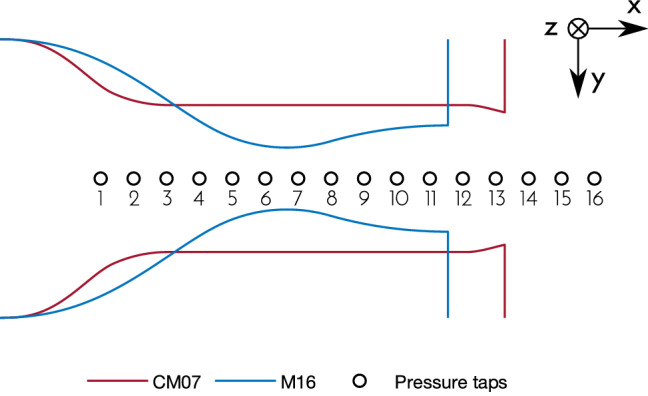
Tested nozzles. CM07 features a large constant area region, designed to achieve a Mach number $M=0.7$. M16 is designed to achieve an outlet Mach number $M=1.6$. Circles (diameter not in scale) indicate the position of pressure taps along the nozzle axis

In the test section, the nozzle is obtained through a pair of steel contoured walls, shaped according to the test conditions. The frontal wall is provided by a planar quartz window, which represents the optical access, while in the rear steel plate a series of 0.3 mm pressure taps is machined. Both the test section and the plenum are electrically heated to avoid vapor condensation. Figure 1 reports a sketch of the two nozzle profiles employed as well as the location of pressure taps (diameter not in scale).

The nozzle characteristic time is more than two orders of magnitude lower than the HPV emptying process time (Spinelli et al. 2013; Pini et al. 2011), and thus the investigated flow can be regarded as a quasi-steady state one at each time instant. Therefore, several steady flow fields at different times and levels of non-ideality can be conveniently extracted from data acquired during the unsteady operation of the facility, since the employed instrumentation exhibits a sufficiently high-frequency response. As a practical measure for the level of non-ideality, the compressibility factor in stagnation conditions $Z_T$ is used, where the compressibility factor is $Z = Pv/RT$, with *v* specific volume and *R* gas constant. A more detailed description can be found in Spinelli et al. (2018).

Total temperature $T_T$ and total pressure $P_T$ are measured in the plenum and define the total condition of the nozzle flow under scrutiny. Two different thermocouples of type K and type J are employed, both located at the plenum axis.

Due to current unavailability of calibrated pressure probes for non-ideal flows, static pressure *P* is measured along the nozzle axis through wall taps, as well as total pressure $P_T$, which is acquired at the plenum wall due to the negligible velocity of the local flow ($\sim \,{1}\,{\mathrm{m}/\mathrm{s}}$). Wall taps are connected to pressure sensors via pneumatic lines directly machined either at the plenum wall or within the test section rear plate. Miniaturized piezoresistive transducers with a sensing element diameter of 3.8 mm and maximum working temperature of ${454}\,{^{\circ }\mathrm{C}}$ are employed. The flow temperature significantly differs from the ambient one, and its variability is not negligible during a test; therefore, the considerable sensitivity to temperature of transducers required their calibration at different operating temperatures, obtaining a voltage $V_P$, pressure *P*, temperature *T* calibration surface. An on-line zero procedure is carried out before each test run using a high precision barometer, to compensate for possible zero drift of the calibrating surface. All these uncertainty contributions are considered in the formulation of the combined pressure uncertainty.

## Experiments and nozzle design

To assess the accuracy of direct velocity measurements in a non-ideal vapor flow of a molecularly complex fluid, a test campaign with different nozzles and thermodynamic conditions was planned. A converging and a converging-diverging nozzle were considered, both with constant thickness (planar, two-dimensional nozzles). The feasibility of direct velocity measurements in the TROVA by means of LDV was first assessed in a subsonic flow, thus at relatively moderate velocity, and in a region of low or no velocity gradient. Then, a supersonic flow was characterized, both in regions of high and almost zero velocity gradient. Therefore, two different nozzles were employed, whose characteristics are reported in Table 1.

**Table 1:** Characteristics of nozzles designed for direct velocity measurement tests

Nozzle id	Fluid	$M_{\text {des}}$	$P_T\,\mathrm{(bar)}$	$T_T\,^{\circ }\mathrm{C}$	$r_t/H$	$H \mathrm{({mm})}$
CM07	MM	0.7	5	210	sharp	17.1
M16	MM	1.6	21.4	254	5	8

The nozzle name, working fluid, total conditions $P_T, T_T$ at design operating point, the non-dimensional curvature $r_t/H$ at the throat, and the throat semi-height *H* are reported. For the converging nozzle CM07, the design Mach number $M_{\text {des}}$ refers to the constant area portion, while for the converging-diverging nozzle M16, it refers to the outlet section

The first, called CM07, is a converging nozzle, where the flow is expanded and accelerated up to sonic conditions at the nozzle outlet, which corresponds to the geometrical throat. The nozzle profile is sketched in Fig. 1. It features a large portion at constant cross-sectional area. This region is designed to obtain Mach number $M\approx 0.7$ with low velocity gradient. Indeed, the flow slightly expands due to a limited growth of the wall boundary layer.

The first converging portion profile is obtained through a 5th-order polynomial, while the second one is composed by a straight segment connected to the constant area region through a cubic spline. The design of both converging portions is made so to avoid possible separation bubbles or excessive gradients.

The outlet section was designed with sharp edge, so to set the geometrical throat. The outlet semi-height is $H={17.1}\,{\mathrm{mm}}$, while the constant area semi-height is $Y_c={19}\,{\mathrm{mm}}$, thus resulting in the area ratio $H/Y_c=A_t/A_c=0.9$ needed to achieve Mach number $M=0.7$ at design conditions for MM (see Table 1); $A_t$ is the throat area and $A_c$ the area of the zero gradient region. The design total conditions are $P_T={5}\,{\mathrm{bar}}$ and $T_T= {210}\,{^{\circ }\mathrm{C}}$ with siloxane MM. It is worth noticing that the nozzle operates in choked and under-expanded conditions; moreover, due to the significant change in total conditions it almost always operates off-design.

The second nozzle (Fig. 1) is of the converging-diverging type. It is called M16 and is used to perform measurements in the supersonic regime in both a region of almost zero velocity gradient and a region of high velocity gradient.

The diverging portion of the nozzle was designed using the method of characteristics implemented with state-of-the art thermodynamic models able to capture the non-ideal behavior of the flow (Guardone et al. 2013). A first portion of the diverging section is a circular arc, that provides an expansion to the desired outlet pressure value. Downstream, the flow is then deviated to obtain a uniform profile at the outlet in the so-called turning region, designed imposing mass conservation. A 5th-order polynomial was used for the nozzle profile of the converging part. The two portions of nozzle profiles are connected by imposing the same first and second derivatives at the nozzle throat.

M16 is designed for a uniform outlet Mach number $M=1.6$ at $P_T={21.4}\,{\mathrm{bar}}$ and $T_T={254}\,{^{\circ }\mathrm{C}}$ with siloxane MM. The throat semi-height is $H={8}\,{\mathrm{mm}}$, while the non-dimensional curvature is $r_t/H=5$. Also in this case, the nozzle normally operates off-design, and thus the flow at outlet is not perfectly uniform and the delivered Mach number differs from $M=1.6$, being typically higher at lower $P_T$.

Tests span from strongly non-ideal conditions to almost ideal ones. A single velocity measurement point along the nozzle axis is taken for each run. Indeed, the blow-down operation of the facility entails time-dependent total conditions, as well as measured pressure ratio due to flow non-ideality, and velocity. Rather than traversing the nozzle, it was preferred to take measurements at one location only for the whole test duration, in order to observe, at one measurement point, the flow field evolution across the maximum span of non-ideality level. The condition at lowest total compressibility factor encountered in this test campaign features $Z_T\approx 0.75$ at $P_T\approx {8.5}\,{\mathrm{bar}}$ and $T_T\approx {208}\,{^{\circ }\mathrm{C}}$. For tests at $M\approx 0.7$ and almost zero velocity gradient, the measurement point is located in the second half of the constant area region, where both CFD calculations and preliminary tests showed an almost uniform flow. The measurement volume is located at the axial coordinate of tap 10 (Fig. 1) and at half depth of the channel. In supersonic tests (nozzle M16), the measurement point is placed just downstream the throat (at tap 8, see Fig. 1), in the expansion region of the nozzle, for the high gradient case, while it is placed in the uniform outlet region (tap 11) for the near-zero gradient case.

## Seeding system and LDV setup

### Particle selection

The LDV technique was chosen in the frame of the presented work and a seeding system was specifically designed. Indeed, seeding must be performed in a high temperature, high pressure and potentially condensing flow without contaminating the carrier flow, thus solid particles are the only viable option. Liquid particles are not suitable for flow seeding at conditions of interest here. Droplets can evaporate or change dimension due to temperature and pressure gradients, or can contaminate the working fluid by mixing. Thus, solid particles are used.

**Table 2:** Properties of the particles considered for seeding in the TROVA

	$\mathrm{TiO}_2$	$\mathrm{SiO}_2$	$\mathrm{Al}_2\mathrm{O}_3$
Density (${\mathrm{kg}/\mathrm{m}^3}$)	3900–4200	2200	3960
Melting point (${^{\circ }\mathrm{C}}$)	1830	1700	2015
Diameter ($\mathrm{nm}$)	150–250	100–150	430
Refractive index	2.6–2.9	1.54	1.79

The selected one is $\mathrm{TiO}_2$

Metallic oxides show high melting point, thus making them a common choice for high temperature applications, such as combustion. Among others, titanium dioxide $({\mathrm{TiO}_2})$, silicon dioxide $({\mathrm{SiO}_2})$ and aluminum oxide $(\mathrm{Al}_2\mathrm{O}_3)$ were selected as candidates for the TROVA flow seeding; Table 2 reports particle properties of significance for LDV applications. All candidate particles feature very high melting point and sub-micrometric diameter. Titanium dioxide and aluminum oxide feature high density, while silicon dioxide is half as dense. Concerning the refractive index, $\mathrm{TiO}_2$ is by far the better option.

The tracing ability of the considered particles can be assessed by solving the Basset–Boussinesq–Oseen (BBO) equation (Albrecht et al. 2003) which considers all forces acting on a particle entrained in a flow. It describes the motion of a spherical particle whose Reynolds number relative to the flow $\mathrm{Re}_p$ is zero. The slip factor $s=\left( V_f-V_p\right) /V_f$ ($V_p$ is the particle velocity and $V_f$ is the fluid velocity) of each particle was estimated along the nozzle axis by simplifying the BBO equation neglecting all terms, but the Stokes drag, which is modified with a $\phi$ coefficient to account for $\mathrm{Re}_p\ne 0$

1 $$\begin{aligned} \frac{\pi }{6} d_{p}^3 \rho _{p} \frac{\mathrm{d}V_p}{\mathrm{d}t}=-3 \pi \mu d_{p} \left( V_p-V_f\right) \phi , \end{aligned}$$

where $d_p$ is the particle diameter, $\rho _p$ is the particle density, and $\mu$ is the viscosity of the fluid. The value of $\phi$ is selected on the basis of the particle Reynolds number. Clift et al. (1978) report correlations for $\phi$ subdivided in ten intervals of $\mathrm{Re}_p$. A solution of the flow in nozzle M16 was computed using the one-dimensional (1D) theory, with total conditions $P_T={8.1}\,{\mathrm{bar}}$ and $T_T= {205}\,{^{\circ }\mathrm{C}}$, which are representative of a typical supersonic non-ideal flow in the test section. Equation (1) was numerically solved by considering the worst case of $V_p={0}\,{\mathrm{m}/\mathrm{s}}$ (i.e., $s=1$) and $V_f$ from 1D calculation as boundary condition at nozzle inlet. The computed slip factor is 100% at nozzle inlet, as imposed by the boundary condition, and rapidly decreases below 0.01%, which means that particles adapt very quickly to the fluid velocity. The slip factor then increases and reaches a maximum value in the region of maximum velocity gradient and then decreases again in the uniform region of the nozzle. Maximum slip is slightly below 1% for the case of $\mathrm{Al}_2\mathrm{O}_3$, while is lower than 0.5% and 0.1% for $\mathrm{TiO}_2$ and $\mathrm{SiO}_2$, respectively.

As it was predictable, silicon dioxide exhibits the lowest slip factor profile, due to its low diameter and density, the highest slip factor is shown by aluminum oxide, while titanium dioxide lies in between. Almost one order of magnitude separates the slip factors of considered particles. However, $\mathrm{SiO}_2$ features also the lowest refractive index (roughly half than the $\mathrm{TiO}_2$ one). This fact, together with the very low particle diameter, poses some doubts on the detectability of $\mathrm{SiO}_2$, considering also that the test section is confined by a quartz window and the quality of the signal may be lowered by the presence of undesired reflections. Since slip factor below 0.5% was considered satisfactory, titanium dioxide was finally chosen, considering its high refractive index and particle diameter that shows a good trade-off between dynamic and optical properties.

### Seeding system design and operation

Flow conditions considered for the design of the TROVA seeding system are maximum pressure $P_T={25}\,{\mathrm{bar}}$ and maximum temperature $T_T={300}\,{^{\circ }\mathrm{C}}$, which make standard seeding devices not suitable, thus requiring a completely customized system.

To avoid working fluid contamination, no auxiliary fluids other than the organic working fluid itself can be used. The principle of the seeding system is to employ a liquid suspension of solid tracer particles in the working fluid, which is injected through an atomizer at high pressure and temperature in the plenum ahead of the test section. The liquid portion of the suspension evaporates, thus releasing the solid particle to be entrained in the flow. A further design constraint is the flow rate of seeding particles required to obtain a mean particle number in the measurement volume lower than 0.1. Indeed, this value permits to achieve a single particle signal with a probability of 99.5% (Albrecht et al. 2003) preserving high signal quality and data rate.

**Fig. 2:**
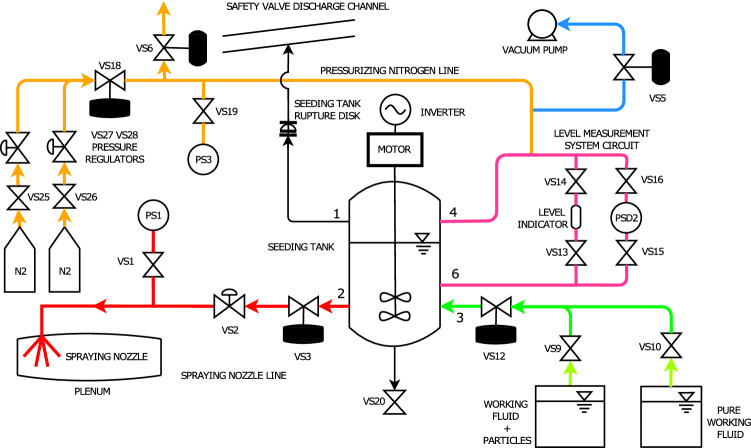
Schematic of the seeding system employed in the TROVA

Different options were considered to inject the suspension into the main flow, including the use of a syringe pump coupled with an ultrasonic atomizer, which was discarded, due to its high cost and uncertainties in the operation of the atomizer. The final choice was a system based on a tank, filled with the liquid suspension to be atomized, pressurized with nitrogen, to achieve the pressure required for injection in the plenum. Figure 2 reports a schematic of the final design of the system. It features five parts, represented with different colors.

The seeding tank is designed to withstand a pressure of ${50}\,{\mathrm{bar}}$ at ambient temperature, which is the operating one. It contains the suspension, whose homogeneous concentration is guaranteed by a propeller stirrer. The suspension, pressurized with nitrogen, is injected into the plenum upstream of the test section using an atomizing nozzle, which is coaxial with the test section and oriented as the main flow. The atomizer is of full cone type, featuring an aperture angle of ${30}{^{\circ }}$, and its operating pressure is regulated in order obtain the desired flow rate of suspension. A trigger signal from the controller computer starts the test and data acquisition, as well as the injection of the suspension.

The mass flow rate of the injected suspension is two to three orders of magnitude lower than the flow rate of the main flow. Therefore, the influence of liquid injection on the main flow properties was considered negligible, as pointed out by both mixing calculation (under the equilibrium hypothesis) and by preliminary tests performed with and without liquid injection, which showed unaltered pressure distribution along the nozzle axis.

The $\mathrm{TiO}_2$ particles were selected according to data provided by the manufacturer (in particular on clustered particle dimension of ${200}\,\mathrm{nm}$, while primer particles are ${20}\,\mathrm{nm}$). Though unlikely, considering the seeding system implemented and the results described in the following, particle agglomeration cannot be completely ruled out. An experimental estimation of the dimension of particles actually seeding the flow was not performed in this work and is of certain interest as a future development. Possible agglomeration would impact high velocity gradient regions; however, the maximum value selected for the calculated slip factor (0.5%) was considered sufficiently low to make limited agglomeration acceptable without compromising the accuracy of LDV measurements in such regions.

For a detailed description of the seeding system and of its operation, the reader is referred to Gallarini (2020).

### LDV system and experimental setup

The LDV setup is a two-component back-scattering system, which employs two ${1}\,{\mathrm{W}}$ diode-pumped solid-state lasers. The two laser beams exhibit a diameter of about ${1}\,{\mathrm{mm}}$ and a wavelength of ${489.5}\,{\mathrm{nm}}$ and ${513.9}\,{\mathrm{nm}}$, respectively. To avoid directional ambiguity a ${40}\,{\mathrm{MHz}}$ frequency shift is applied on each couple of laser beams.

Ahead of the focusing length, a beam expander with ratio $E=1.874$ increases both beam diameter and distance, thus leading to a decreased measurement volume size. The adopted focusing lens features a focal length $F={310}\,{\mathrm{mm}}$. The measurement volume size, evaluated according to Albrecht et al. (2003), resulted in $2a_0\approx {0.1}\,{\mathrm{mm}}$ and $2b_0\approx {0.1}\,{\mathrm{mm}}$ in *x* and *y* coordinates (coincident with the nozzle axis and height, see the coordinate system of Fig. 1), while $2c_0\approx {0.9}\,{\mathrm{mm}}$ in the third direction *z* (the nozzle depth); $a_0$, $b_0$, and $c_0$, represent the ellipsoidal measurement volume semi-axis length in *x*, *y*, and *z* directions, respectively.

**Fig. 3:**
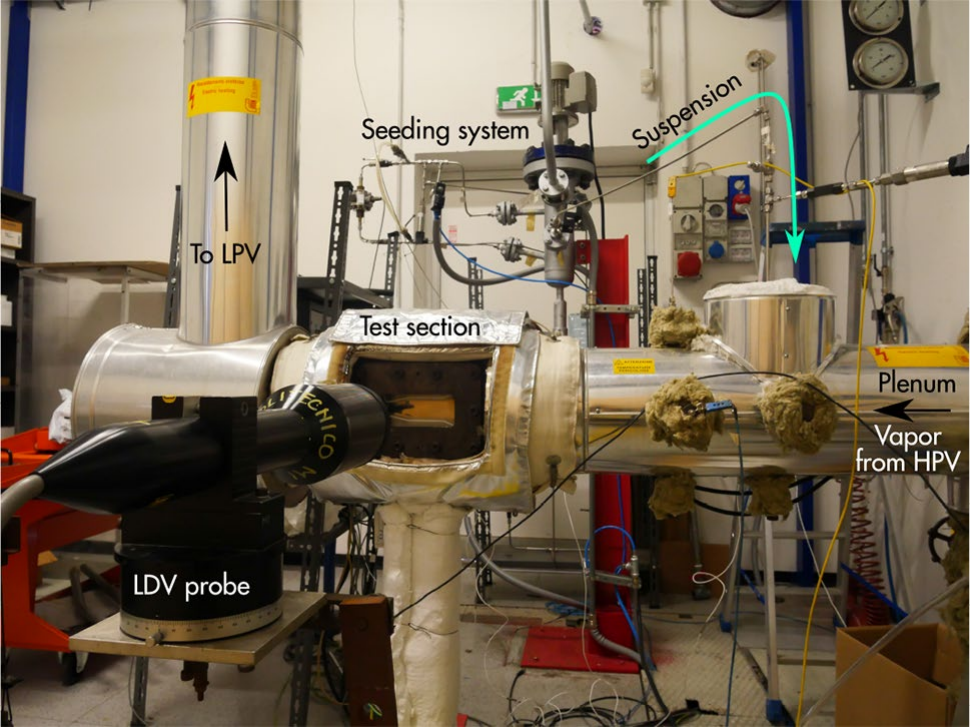
TROVA test section and LDV setup. On the left, the LDV probe is positioned in front of the test section, accommodating the nozzle. The test section is fed from right to left through the plenum (right) and discharges the vapor into the low pressure vessel through the pipe on the left. In the background, the seeding system is visible

The burst spectrum analyzer is a Dantec F 800, which features a maximum input frequency of ${200}\,{\mathrm{MHz}}$. The optical setup described above allows velocity measurements up to about ${400}\,{\mathrm{m}/\mathrm{s}}$. Synchronization with external sources is assured by external trigger inputs. Figure 3 shows an overview of the experimental setup; the flow in the nozzle test section is from right to left.

## Processing of velocity data

Tests were carried out acquiring pressure, temperature and velocity signals, which were synchronized through a trigger signal from the TROVA control computer. The LDV data set features six variables: *x* and *y* velocity components $\tilde{V}_{x}$ and $\tilde{V}_{y}$, particle arrival times $\tilde{t}_{x}$ and $\tilde{t}_{y}$ and transit times $\tilde{tt}_{x}$ and $\tilde{tt}_{y}$. The order of magnitude of data rate ranges from 10$^2$ to $10^3\,\mathrm{Hz}$.

Due to the blow-down operation of the TROVA, total conditions of the nozzle continuously change in time, resulting in a time-dependent velocity field. Thus, the statistical analysis is carried out on contiguous non-overlapping blocks of ${1}\,{\mathrm{s}}$ width. This provides a sufficient number of signals for the statistical analysis, while keeping the mean velocity variation in time within the interval approximately below 0.5%. Centered time intervals are adopted $\left[ t-{0.5}\,{\mathrm{s}};t+{0.5}\,{\mathrm{s}}\right]$ to further reduce the approximation introduced by the batch operation.

To tackle the issue of the velocity bias introduced by the arithmetic mean, the transit time weighted mean is implemented, which is the recommended choice if transit time is available (Albrecht et al. 2003). Thus, mean velocity is computed according to Eq. (2), where $\hat{V}_{x,i}$ and $\hat{tt}_{x,i}$ are, respectively, the velocity observations and the transit time in the $k-th$ time interval

2 $$\begin{aligned} V_{x,k}= \frac{\sum _{i \in k} \hat{tt}_{x,i} \cdot \hat{V}_{x,i}}{\sum _{i \in k} \hat{tt}_{x,i}}, \end{aligned}$$

and similarly for the *y* component.

Diverse options are available in the open literature to define the standard deviation of the weighted average. Some relations, each with some drawbacks, were proposed in literature and Gatz and Smith (1995) reported an assessment of their accuracy, using the bootstrap method as a benchmark. This method is a computer-based technique which permits to compute the accuracy of statistical estimates, without making parametric assumptions on the distribution underlying the data (see the book by Efron and Tibshirani 1994 for details). It is particularly useful in this case, where the distribution function of LDV samples is not known and a weighted average is being used. To avoid possible drawbacks of available relations for the standard deviation of the weighted mean and due to the very low computer power required to analyze the LDV data set, the bootstrap method was directly implemented.

The obtained standard deviation interval is taken as the contribution of the dispersion of data to the 95% confidence level uncertainty of the LDV measurement. This method was applied to both the *x* and *y* velocity component separately, to obtain the profile of average velocity and its dispersion as a function of test time.

Other uncertainty sources may arise from mean velocity gradients normal to the direction of the component being measured. It can be proved (Albrecht et al. 2003) that the mean *x* velocity averaged over the measurement volume $\bar{V}_{x,MV}$ can be approximated as

3 $$\begin{aligned} \bar{V}_{x,MV} = \bar{V}_{x}\left( y_c\right) +\underbrace{\frac{b_d^2}{8}\frac{d^2 \bar{V}_x\left( y\right) }{dy^2}\Big |_{y=y_c}}_{\text {velocity error}}, \end{aligned}$$

where $\bar{V}_{x}\left( y\right)$ is the mean *x* velocity, $y_c$ is the *y* coordinate of the center of the measurement volume, and $b_d$ is the ellipsoid semi-axis in the *y* direction. Here, the error contribution was estimated from velocity profiles extracted at the nozzle axis from CFD calculations (which allowed to compute the second order derivative appearing in last term in Eq. (3)) and resulted of the order of $1\times 10^{-4}\mathrm{m}/\mathrm{s}$ in the worst case; thus, it was considered negligible.

During a test, density undergoes a significant variation, and thus the refractive index *n* in the measurement point changes as a result of the Gladstone–Dale equation (Eq. (4))

4 $$\begin{aligned} n=1+k\rho . \end{aligned}$$

where *k* is the Gladstone–Dale coefficient. The angle $\Theta$ between the two beams changes accordingly, thus causing a slight movement of the measurement volume in the direction of positive *z*, toward the rear plate. This displacement was estimated for all performed tests, obtaining a maximum value of 1.1% of the nozzle semi-depth, that is considered negligible, due to the planar nature of the flow.

The last considered source of error is the effect of a gradient of refractive index of the vapor. Indeed, due to a density gradient, laser beams can be deflected, leading to both a displacing and rotation of the measurement volume. In the laser Doppler velocimetry case, beams enter the perturbed region with a non-negligible angle in the plane of laser beams with respect to the density gradient.

Given that *x*, *y*, *z* are the directions of the nozzle axis, height and depth, respectively, the displacement of the measurement volume in the streamwise direction is given by

5 $$\begin{aligned} x_{2}-\left( x_{2}\right) _{\partial n/\partial x=0}=\frac{L^2}{2}\frac{1}{n}\frac{\partial n}{\partial x}. \end{aligned}$$

where subscript 2 refers to the nozzle axis and *L* is the nozzle semi-depth.

The value of *n* to be used in previous calculations can be obtained from Eq. (4), if the Gladstone–Dale coefficient *k* for MM is known. Under the hypothesis that the molecular polarizability is a weak function of temperature and pressure, the refractive index of MM can be obtained from the Lorentz-Lorenz relation (Liu and Daum 2008) as

6 $$\begin{aligned} n=\sqrt{\frac{M_m+2\rho R_M}{M_m-\rho R_M}}, \end{aligned}$$

where $R_M=\frac{\left( n^2-1\right) M_m}{\left( n^2+2\right) \rho }$ is the molar refraction, $\rho$ is the material density, and $M_m$ is the molar mass. This equation, for low densities, is well approximated by a linear relation between *n* and $\rho$. A Gladstone–Dale constant $k_{\mathrm{MM}}={4.5\times 10^{-4}}\,{\mathrm{m}^3/\mathrm{kg}}$ was found by applying the least squares method to values obtained from Eq. (6) with $R_M$ computed with a refractive index of liquid MM $n_{\mathrm{MM},\mathrm{liq}}=1.3772$ (Wohlfarth 2017).

The sources of error arising from refractive index gradients were evaluated for the case of maximum gradient using data from CFD simulations (see 6.3), yielding a maximum angular deviation $|\epsilon |={0.18}{^{\circ }}$ and beam displacement $x_{2}-\left( x_{2}\right) _{\partial n/\partial x=0}={14}\,{\upmu \mathrm{m}}$. The error from the angular deviation of the velocity component being measured (the apparent velocity) with respect to the *x* axis is negligible, being approximately $5\cdot 10^{-4}{\%}$. The displacement of the beam leads to an error which depends on the velocity gradient at the measurement point and was estimated to be ${0.07}\,{\mathrm{m}/\mathrm{s}}$ with respect to a mean velocity of $\approx \,{180}\,{\mathrm{m}/\mathrm{s}}$. Further $x_{2}-\left( x_{2}\right) _{\partial n/\partial x=0}$ is to be compared to the diameter of pressure taps, which is ${300}\,{\upmu \mathrm{m}}$, thus the displacement is lower than the resolution of pressure measurements. For the aforementioned reasons, the contribution of density gradient on measurement uncertainty was neglected.

Summarizing, the only considered contribution to velocity measurement uncertainty is the one originating from the scatter of velocity samples. In case of measurements in high axial velocity gradient regions, the contribution to the uncertainty related to the measurement volume positioning was also considered, referring to a position uncertainty of ${0.5}\,{\mathrm{mm}}$ along the nozzle axis. Contrarily, the contribution related to positioning in the plane normal to the nozzle axis was not considered due to negligible velocity gradients. The expanded uncertainty (95% confidence level) for the mean velocity, resulted of the order of 0.1–0.2% for measurement taken within almost uniform flows, while it reaches the order of 1–2% in the case of high velocity gradient regions.

## Expanding flow characterization: direct velocity measurement results

In this section, results of the complete characterization of non-ideal compressible fluid flows by means of pressure, temperature and direct velocity measurements are reported and thoroughly discussed. The flow is characterized in a single measurement point on the nozzle axis. Three different cases are analyzed, a subsonic flow at $M\approx 0.7$, with near-zero velocity gradient (Sect. 6.1), a supersonic flow at $M\approx 1.7$, in a region at near-zero velocity gradient (Sect. 6.2), and a supersonic flow at $M\approx 1.4$, in a region at high velocity gradient (Sect. 6.3).

### Subsonic non-accelerating flow

Three tests were chosen for consistency and repeatability assessment. Table 3 reports the most non-ideal thermodynamic conditions of each test, corresponding to minimum total compressibility factor $Z_T$, and the most ideal one, in dilute-gas conditions corresponding to $Z\approx 1$; the nozzle employed is the converging one CM07. From now on, the notation [Nozzle name].[Test id] is used to refer to a test, while keeping clear the type of nozzle.

**Table 3:** Thermodynamic conditions of tests carried out on the nozzle CM07 with siloxane MM

Test id	Nozzle	Min. $Z_T$ condition			Max. $Z_T$ condition		
		$P_T$ $({\mathrm{bar}})$	$T_T$ $(^{\circ }\mathrm{C})$	$Z_T$ (–)	$P_T$ $({\mathrm{bar}})$	$T_T$ $({^{\circ }\mathrm{C}})$	$Z_T$ (–)
78	CM07	7.385	218	0.807	0.823	202	0.979
79	CM07	6.914	217	0.820	0.714	204	0.982
81	CM07	7.517	215	0.799	0.976	200	0.975

Total pressure $P_T$, total temperature $T_T$, and total compressibility factor $Z_T(T_T,P_T)$ are reported for the most non-ideal condition and for the most ideal one

Total conditions were chosen to meet the desired minimum value of total compressibility factor, which was set at $Z_T\lesssim 0.8$. A further constraint is, of course, the thermal stability of the working fluid. The moderate level of non-ideality permits to have maximum temperatures always below ${220}\,{^{\circ }\mathrm{C}}$, which are totally safe from the thermal stability point of view (Gallarini et al. 2019). The explored thermodynamic region is quite large and goes from slightly superheated non-ideal flows with $Z_T\approx 0.8$ to almost ideal gas flows with $Z_T\approx 0.98$.

After a series of preliminary tests, the pressure gradient in the nozzle axis direction $\partial P / \partial x$ in the region between pressure taps $P_9$ and $P_{11}$ resulted to be the lowest; thus, the location of pressure tap 10 was chosen as measurement point.

The LDV measurement volume was placed with the direction of the two measured velocity components aligned with the nozzle *x* and *y* axes. The measurement volume was positioned at the center of the channel, in both *y* and *z* directions. No velocity component is measured along nozzle depth direction *z*.

Various tests were carried out and compared in terms of measured total pressure, total temperature, static pressure and velocity. The repeatability and consistency of measurements was considered very good, even if uncertainty bars of each test run do not always overlap. Indeed, a perfect repeatability of test total conditions cannot be achieved due to uncertainties related to the mass loaded and to possible temperature stratification in the HPV.

Test CM07.81 is now taken as representative of the campaign and is discussed in detail.

**Fig. 4:**
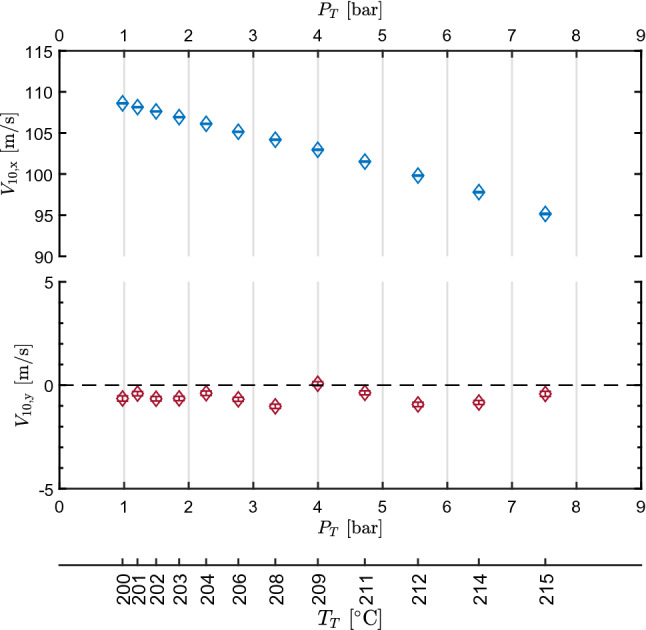
Measured *x* and *y* velocity components at tap 10 as a function of total pressure $P_T$ and total temperature $T_T$ for the test CM07.81

The bootstrap method was applied to compute the mean *x* and *y* velocity components at tap 10 ($V_{10,x}$ and $V_{10,y}$) as a function of test time, which corresponds to different $P_T$ and $T_T$. Figure 4 reports the resulting velocity components as a function of total conditions; total pressure $P_T$ is reported on the first horizontal axis, while a second one reports the corresponding total temperature $T_T$. The *y*-velocity component is approximately zero, while $V_{10,x}$ is of the order of ${100}\,{\mathrm{m}/\mathrm{s}}$, thus proving the correct alignment and positioning of the probe. Considering the two orders of magnitude difference between the *x* and *y* components, from now on the velocity magnitude is always taken equal to the *x* velocity component, being the deviation introduced below measurement uncertainty.

Static-to-total pressure ratio and velocity as a function of total conditions are analyzed. Experimental quantities are compared to those extracted at the measurement point from a two-dimensional (2D) viscous CFD calculation, performed using ANSYS$^{\textregistered }$ Fluent, Release 19.2. The flow symmetry with respect to the nozzle *x* axis is exploited; the computational domain is composed by the converging nozzle with a sudden enlargement after the geometrical throat. In the case of a converging nozzle, the region where the flow is discharged must also be modeled. Indeed, in this case, at the nozzle outlet (geometrical throat) the flow is still slightly subsonic, and thus the downstream enlargement is needed to reach $M=1$ and define a sonic line, as happens in the real condition occurring in the TROVA. A hybrid mesh was adopted, with quadrilateral cells and proper refinement in the proximity of the wall, for a correct solution of the boundary layer. The size of the first cell adjacent to the wall was set to result in a dimensionless wall distance $y^+\approx 1$. Four meshes of increasing number of elements were considered, and the finest one (${1.92 \times 10^5}$ elements) was finally employed, due to acceptable computational time. The grid convergence was considered satisfactory since the deviations between results obtained with the two finest meshes were below 0.05% for pressure and 0.1% for velocity. An implicit, density-based solver implementing a Roe flux-difference splitting was employed with a 2nd order upwind spatial discretization for convective fluxes. The employed turbulence model is $k-\omega$ SST, while the thermodynamic model is the Helmholtz energy fundamental relation for MM by Thol et al. (2016) implemented in RefProp.

For a comparison with LDV data, the flow velocity can be computed also from total conditions and the measured static pressure, by resorting to the isentropic expansion hypothesis.

**Fig. 5:**
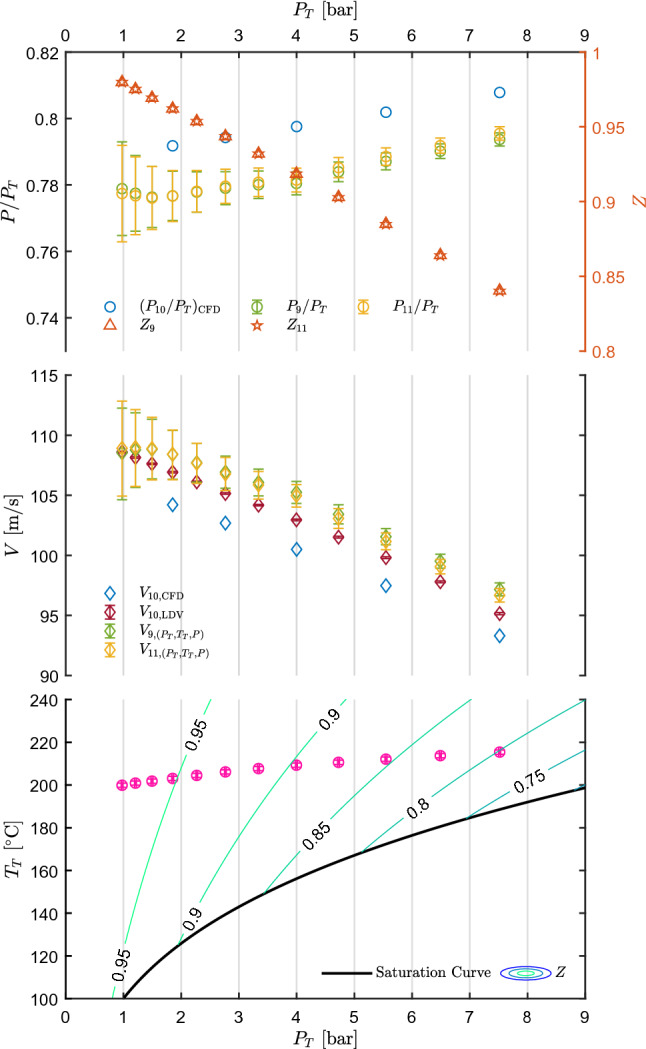
Results of test CM07.81. Top left axis: static-to-total pressure ratio at the nozzle axis measured at tap 9 and 11, $P_9/P_T$ and $P_{11}/P_T$, as a function of measured total pressure $P_T$. Pressure ratio extracted at tap 10 from CFD, $(P_{10}/P_T)_{\text {CFD}}$, is also plotted. Top right axis: compressibility factor at the measurement points. Center: velocity measured at the nozzle axis in correspondence of tap 10, $V_{10,\text {LDV}}$, as a function of $P_T$ along with velocity obtained at taps 9 and 11 through the thermodynamic model and $P_T$, $T_T$, *P* measurements, $V_{9,(P_T,T_T,P)}$ and $V_{11,(P_T,T_T,P)}$. Velocity extracted at the measurement point from CFD, $V_{10,\text {CFD}}$, is also plotted. Bottom: measured total temperature $T_T$ as a function of total pressure $P_T$, along with the saturation curve of MM and superimposed to contours of compressibility factor *Z*. All uncertainty bars correspond to 95% confidence level

Figure 5 shows the results of test CM07.81. The bottom graph reports the total conditions $P_T$ and $T_T$ along with the MM saturation curve and compressibility factor *Z* contours. This graph shows the extent of the explored thermodynamic region and is the reference basis for the central and top graph. The central chart shows the measured velocity along with those calculated from CFD simulations and from total conditions and the measured static pressure under the isentropic hypothesis, as a function of total pressure $P_T$. Each point in the velocity graph corresponds to a point in the total conditions graph, where the relation with total pressure, total temperature and total compressibility factor can be appreciated. Pressure was measured at taps 9 and 11, and thus two velocity values are reported. The velocity extracted from the CFD calculation refers to LDV measurement, at axial location corresponding to tap 10. The top graph reports the static-to-total pressure ratio measured and extracted from CFD. The right axis reports the compressibility factor at the measurement points, computed from total conditions and the measured static pressure.

The pressure ratio at the two selected taps is almost the same, thus confirming the non-accelerating behavior of the flow in this region. As expansions starting from more ideal states (increasing $Z_T$) are considered, decreasing $P/P_T$ is observed, accordingly with the 1D theory and previous experiments on non-ideal expanding flows (Spinelli et al. 2018). Velocity increases as dilute conditions are approached (i.e., moving from right to left in Fig. 5), contrarily to the behavior expected for an ideal gas expansion occurring at slightly reducing total temperature, as it is the typical case of tests performed on the TROVA and of test CM07.81 here analyzed. The observed velocity increase is therefore to be ascribed to non-ideal effects, as it is predicted by theory. Indeed, at non-ideal gas states the speed of sound is lower than its ideal counterpart due to the effect of repulsive and attractive forces (Colonna and Guardone 2006). Moving from dense gas states to ideal gas ones, the negative contribution to the speed of sound from attractive molecular forces and the positive one from repulsive forces vanishes, thus increasing the values of speed of sound. Mach number also increases, as it was observed in Spinelli et al. (2018), thus leading to an increasing velocity.

Pressure ratio and velocity trends are well captured, as they are consistent with theory and previous experiments. The accordance between measurements and computed values is evaluated through percentage differences defined as

7 $$\begin{aligned} d_{\frac{P}{P_T}\%}\left( \frac{P}{P_T}\right) =\frac{\left( \frac{P}{P_T}\right) _{\mathrm{CFD}} -\left( \frac{P}{P_T}\right) }{\left( \frac{P}{P_T}\right) }\cdot 100, \end{aligned}$$

and

8 $$\begin{aligned} d_{V\%}=\frac{V_j-V_{\text {LDV}}}{V_{\text {LDV}}}\cdot 100, \end{aligned}$$

where $V_{\text {LDV}}$ refers to the directly measured mean velocity, while $V_j$ refers either to the velocity $V_{(P_T,T_T,P)}$ inferred from $P_T, T_T, P$ measurements or to the one extracted from CFD, $V_{\text {CFD}}$. Similarly, subscript $\text {CFD}$ and no subscript refer to CFD and measured pressure ratios, respectively. The related uncertainty is obtained as

9 $$\begin{aligned} \begin{aligned} u_{d_{\frac{P}{P_T}\%}}\left( \frac{P}{P_T}\right)&=\frac{100}{\left( \frac{P}{P_T}\right) }\cdot \\&\quad \cdot \sqrt{\left( \frac{\left( \frac{P}{P_T}\right) _{\mathrm{CFD}}}{\left( \frac{P}{P_T}\right) }\right) ^2\cdot \left( u\left( \frac{P}{P_T}\right) \right) ^2}, \end{aligned} \end{aligned}$$

and, assuming $V_j$ and $V_{\text {LDV}}$ to be uncorrelated, being obtained by independent techniques

10 $$\begin{aligned} \begin{aligned} u_{d_{V\%}}&=\frac{100}{V_\text {LDV}}\cdot \\&\quad \cdot \sqrt{\left( u\left( V_j\right) \right) ^2+\left( \frac{V_j}{V_\text {LDV}}\right) ^2\cdot \left( u\left( V_\text {LDV}\right) \right) ^2}. \end{aligned} \end{aligned}$$

No uncertainty is considered for values extracted from CFD. $u\left( V_{\text {LDV}}\right)$ corresponds to the one computed from the bootstrap technique applied to LDV data. In the case of velocity obtained from $P_T, T_T, P$, the uncertainty was computed with the Monte Carlo method, using the above primary measurement uncertainties as input.

**Fig. 6:**
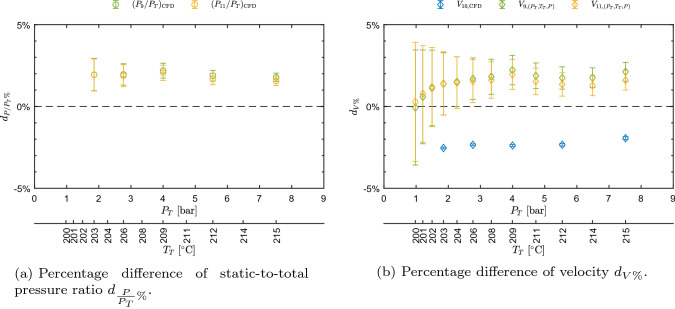
Percentage difference of pressure ratio and velocity among values measured, extracted from CFD, and obtained from $P_T, T_T, P$ measurements, as a function of total conditions $P_T$ and $T_T$ for test CM07.81

Figure 6a shows $d_{\frac{P}{P_T}\%}$ as a function of total conditions $P_T$ and $T_T$. The agreement is very good, as the percentage deviation is approximately around 2.2% for each considered condition. $d_{V\%}$ is reported in Fig. 6b and shows a very good agreement between LDV measurements and both CFD and values computed from pressure and temperature. The deviation is approximately below 2% also in this case. The good accordance between LDV and computed values from $P_T, T_T, P$ proves the consistency between LDV and both pressure and temperature measurements. Despite the good accordance, pressure ratios extracted from CFD appear to be systematically higher than the measured one and CFD computed velocities are lower than the measured one. This can be reasonably ascribed to the boundary layer growth on the front and back walls of the constant area region, which is not accounted for in the 2D simulations. Further investigations on this point are ongoing; however, they are beyond the scope of this paper. Summarizing, the accordance between measurements and computed values is very good, with deviations of the order of 2%; also, measured trends of both $P/P_T$ and *V* prove the non-ideal behavior of the flow.

### Supersonic non-accelerating flow

After the feasibility of laser Doppler velocimetry in a subsonic non-accelerating flow was proved, the following step was the characterization of a supersonic non-accelerating flow, thus entailing a higher velocity to be measured. Therefore, the nozzle employed in this campaign is M16. Various tests were performed and thermodynamic conditions of two of them are reported in Table 4. The two extreme cases of minimum and maximum total compressibility factor are reported, showing that expansions featuring $0.75\lesssim Z_T \lesssim 0.96$ were observed. The maximum temperature is approximately ${210}\,{^{\circ }\mathrm{C}}$, which is far below the region where thermal decomposition can possibly occur.

**Table 4:** Thermodynamic conditions of tests carried out on the nozzle M16 in correspondence of tap 11 with siloxane MM

Test id	Nozzle	Min. $Z_T$ condition			Max. $Z_T$ condition		
		$P_T$ $(\mathrm{bar})$	$T_T$ $({^{\circ }\mathrm{C}})$	$Z_T$ (–)	$P_T$ $(\mathrm{bar})$	$T_T$ $({^{\circ }\mathrm{C}})$	$Z_T$ (–)
93	M16	8.277	206	0.749	1.330	197	0.964
94	M16	8.400	207	0.749	1.528	197	0.959

Total pressure $P_T$, total temperature $T_T$, and total compressibility factor $Z_T(T_T,P_T)$ are reported for the most non-ideal condition and for the most ideal one

The chosen measurement point is in correspondence of tap 11, which is the last tap upstream of the nozzle outlet in the almost uniform zone. The configuration of the LDV system is the same as for the subsonic tests. Pressure is measured at the same position where velocity measurements are taken (tap 11). Also in this case the consistency and repeatability of tests were considered verified.

The test M16.94 is chosen as representative of this condition and is here discussed.

**Fig. 7:**
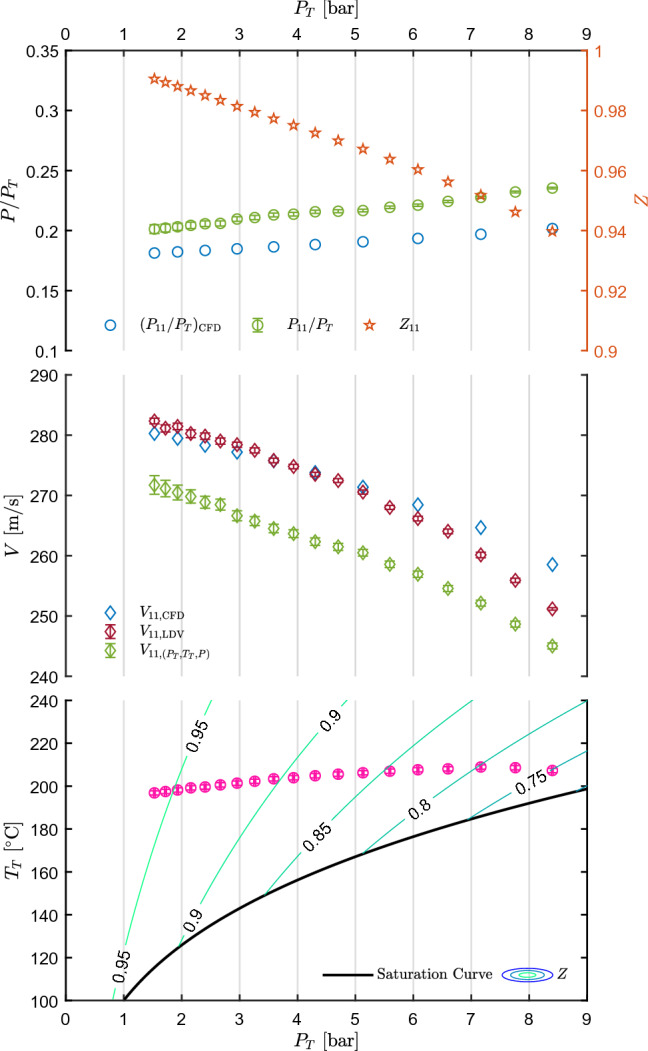
Results of test M16.94. Top left axis: static-to-total pressure ratio at the nozzle axis measured at tap 11, $P_{11}/P_T$, as a function of total pressure $P_T$. Pressure ratio extracted at the measurement point from CFD, $(P_{11}/P_T)_{\text {CFD}}$, is also plotted. Top right axis: compressibility factor at the measurement point. Center: velocity measured at the nozzle axis in correspondence of tap 11 ($V_{11,{\text {LDV}}}$) as a function of $P_T$ along with velocity obtained at tap 11 from $P_T$, $T_T$ and *P* measurements. Velocity extracted at the measurement point from CFD, $V_{11,{\text {CFD}}}$ is also plotted. Bottom: measured total temperature $T_T$ as a function of total pressure $P_T$, along with the saturation curve of MM and superimposed to contours of compressibility factor *Z*. All uncertainty bars correspond to 95% confidence level

Experimental results are shown in Fig. 7 in the same fashion as for the subsonic test. Concerning CFD simulations the employed turbulence and thermodynamic models are the same as for the test with nozzle CM07, while the computational domain was restricted to the converging diverging nozzle. After grid independence analysis, a mesh with ${3.71\times 10^4}$ quadrilateral elements was finally adopted and the wall treatment was the same as for nozzle CM07. Also, the solver, the numerical scheme, the spatial discretization, and the turbulence and thermodynamic models are the same as for nozzle CM07. Both pressure and velocity are only measured at the nozzle axis point corresponding to tap 11, located in the uniform region. Pressure is measured at the wall, while velocity at mid-depth of the channel. The decreasing trend of $P/P_T$ for decreasing total pressure is in accordance with the increase in total compressibility factor $Z_T$, that can be appreciated from the contours of the bottom chart of Fig. 7. The central graph reports the measured velocity, compared to those obtained from the CFD simulation and computed from total conditions and static pressure, by resorting to the isentropic hypothesis. Velocity increases as ideal states are approached, similarly to the case of the subsonic nozzle test.

**Fig. 8:**
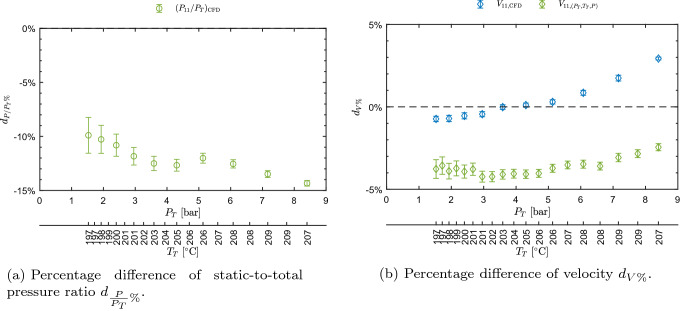
Percentage difference of pressure ratio and velocity among values measured, extracted from CFD, and obtained from $P_T, T_T, P$ measurements, as a function of total conditions $P_T$ and $T_T$ for test M16.94

The percentage difference between CFD and measured static-to-total pressure ratios (Eqs. (7) and (9)) reported in Fig. 8a shows a non-negligible deviation, that is as high as 14.3% in the most non-ideal state and decreases to $\approx 9.9{\%}$ at the highest $Z_T$. Such deviations can be partially ascribed to the boundary layer thickness on the side walls of the channel (*z* direction) in the uniform region, which is not accounted for in the 2D simulations and is consistent with supersonic flow deceleration. Also, at these high velocity levels, possible chamfering of pressure tap edges due to polishing becomes relevant. This would result in an increased measured static pressure due to the recovery of a small fraction of kinetic energy. This is also consistent with a reduction of deviation as the total pressure, and thus kinetic head, reduce. In this respect, it is worth noticing that the maximum deviation of static pressure between measured and CFD data is of about 4.5% of the kinetic head. These analyses are currently underway, but, however, beyond the scope of this paper. However, similar results were observed by Robertson et al. (2019): deviations of $P/P_T$ at the last pressure tap between CFD and experiments were found to be 9.12–23.36% and 6.63–20.65% for Peng–Robinson and Helmholtz energy models, respectively, for a supersonic flow ($M\approx 2$) of R1233zd(E).

Velocity measured by LDV, obtained from $P_T$, $T_T$, *P* data, and computed from CFD simulations were compared (Fig. 8b) by means of percentage deviations with respect to those measured by LDV (Eqs. (8) and (10)). Regarding CFD data, the deviation is maximum ($\approx 2.9$%) at the expansion corresponding to minimum $Z_T$, while reduces to less than 1% at maximum $Z_T$ passing through the zero line. Velocity computed from pressure and temperature measurements deviates from a minimum of $\approx 2.4$% to a maximum of $\approx 4.2$%. In general, the accordance is good between CFD and LDV, while it is worse but still satisfactory between LDV and velocity calculated from $T_T-P_T-P$. It is worth to point out that deviations between CFD and experimental pressure ratios of $\approx 10$–15% lead to a discrepancy of only $\approx 4$–5.5% between CFD and computed velocity.

### Supersonic accelerating flow

The aim of these tests is to characterize the flow and assess the feasibility of laser Doppler velocimetry in a supersonic accelerating flow. Thus, the tested nozzle is M16 and the measurement point for both pressure and velocity is located at the axis in correspondence to tap 8, lying in the expansion region, where pressure and velocity gradients are not negligible. Table 5 reports the thermodynamic conditions at minimum and maximum $Z_T$ during three of the performed tests. The total compressibility factor ranges from a minimum of $Z_T\approx 0.75$ to a maximum of $Z_T\approx 0.97$. Also in this case, a thermodynamic region safe from the thermal stability point of view is explored. The configuration of the LDV system is the same as for the already discussed tests and the measurement volume is located at nozzle mid-depth, while pressure is measured at the wall. Also in this case, the accordance and repeatability of tests are good.

**Table 5:** Thermodynamic conditions of tests carried out on the nozzle M16 with siloxane MM for the measurement of velocity at tap 8, a region of high velocity gradient at Mach number $M\approx1.4$

Test id	Nozzle	Min. $Z_T$ condition			Max. $Z_T$ condition		
		$P_T$ (bar)	$T_T$ $({^{\circ }\mathrm{C}})$	$Z_T$ (–)	$P_T$ $({\mathrm{bar}})$	$T_T$ (${^{\circ }\mathrm{C}}$)	$Z_T$ (–)
101	M16	8.047	210	0.769	1.318	200	0.966
102	M16	8.321	208	0.752	1.488	199	0.960
103	M16	8.134	204	0.750	1.372	197	0.963

Total pressure $P_T$, total temperature $T_T$, and total compressibility factor $Z_T(T_T,P_T)$ are reported for the most non-ideal condition and for the most ideal one

**Fig. 9:**
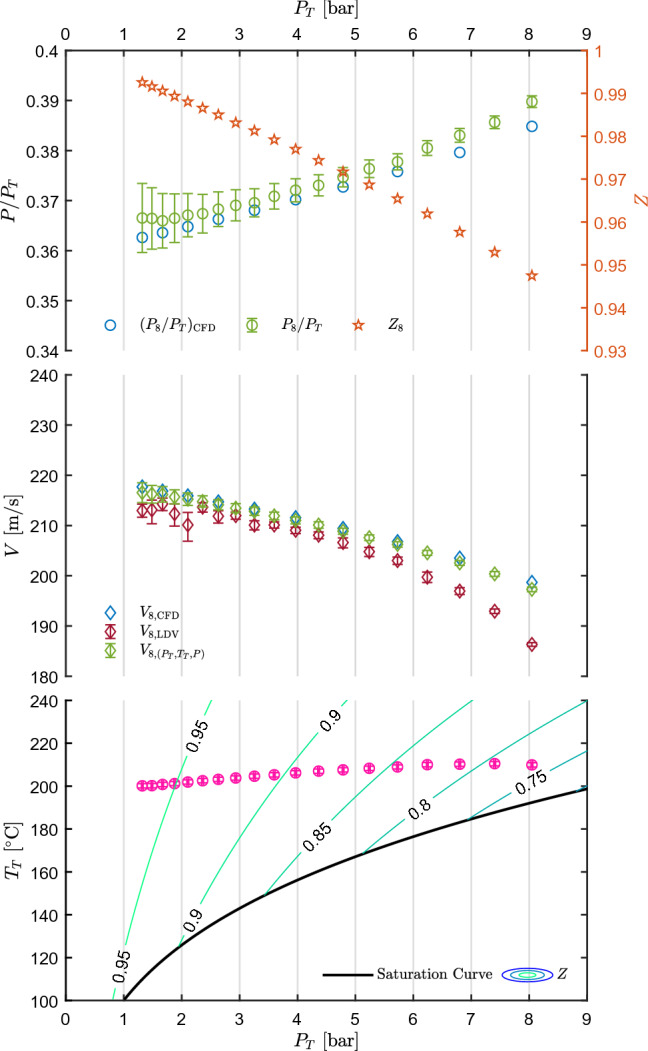
Results of test M16.101. Top left axis: static-to-total pressure ratio at the nozzle axis measured at tap 8, $P_{8}/P_T$, as a function of total pressure $P_T$. Pressure ratio extracted at the measurement point from CFD, $(P_{8}/P_T)_{\text {CFD}}$, is also plotted. Top right axis: compressibility factor at the measurement point. Center: velocity measured at the nozzle axis in correspondence of tap 8, $V_{8,\text {LDV}}$, as a function of $P_T$ along with velocity obtained from $P_T$, $T_T$ and *P*. Velocity extracted at the measurement point from CFD, $V_{8,\text {CFD}}$, is also plotted. Bottom: measured total temperature $T_T$ as a function of total pressure $P_T$, along with the saturation curve of MM and superimposed to contours of compressibility factor *Z*. All uncertainty bars correspond to 95% confidence level

Experimental results of test M16.101 are reported in Fig. 9, along with CFD calculations which were carried out with the already discussed setup (Sect. 6.2). The increase in pressure ratio for increasing non-ideality (decreasing $Z_T$ and increasing $P_T$) is well captured by both measurements and CFD and is in accordance with the 1D theory and all previously discussed results.

The central graph shows the measured velocity $V_8$, compared with the velocity extracted from the CFD simulation and the one computed from total conditions and static pressure. The trend of the three sets of data is similar, but, although the accordance between CFD and calculated velocity is optimal, the measured one shows a non-negligible deviation at high levels of non-ideality. Analyzing the bursts signals, at test start (high pressure), the distribution of measured velocity exhibits a wider scatter toward low velocities with respect to following time instants. The number of low velocity bursts is limited but still sufficient to affect the mean value. Such bursts signal distribution, in the case of an accelerating flow, could be ascribed to the presence of impurities or agglomerated particles sedimented between two consecutive tests within the plenum or the atomizer pipeline and dragged by the main flow during the initial part of the test and resulting in a higher slip. However, this hypothesis requires further verification. Instead, due to the high degree of superheating of the main flow and the extremely low ratio between the injected and main mass flow rates, droplet presence at the measurement point is considered highly unlikely.

The increasing velocity for increasing $Z_T$ is, in this case also, an indication of non-ideality of the flow, resulting from the increase in both Mach number and speed of sound.

**Fig. 10:**
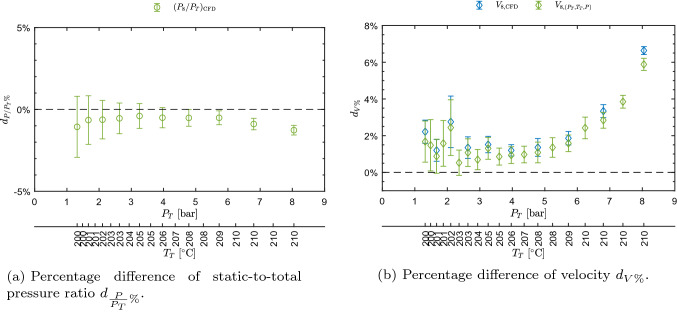
Percentage difference of pressure ratio and velocity among values measured, extracted from CFD, and obtained from $P_T, T_T, P$ measurements, as a function of total conditions $P_T$ and $T_T$ for test M16.101

Figure 10a, b reports the percentage difference of pressure ratios and velocities, respectively, as a function of total conditions, with reference to Eqs. (7)–(10). The accordance among CFD and measurements is very good, in the case of pressure ratios, with discrepancies lower than 1.5%. Regarding velocity, the discrepancy between CFD and computed velocity is very low. LDV instead features a deviation with both CFD and the computed velocity most of the times below 4%, with the exception of the point at the highest pressure with reaches about 6.6%.

Concluding, velocity measured by LDV, obtained from pressure and temperature data via the thermodynamic model, and calculated through CFD simulations exhibits an accordance which is globally considered satisfactory. The behavior at conditions where the maximum discrepancy between velocities is observed needs to be further investigated.

## Conclusions

The first-ever complete characterization of a point in a non-ideal compressible fluid flow was reported. To reach this goal, temperature and pressure measurement were complemented with the first-ever direct velocity measurements in such flows, carried out by means of laser Doppler velocimetry.

The hostile environment represented by the flow to be characterized required the employment of solid materials for flow tracing. Titanium dioxide particles of about ${200}\,\mathrm{nm}$ diameter were selected as the best compromise between dynamic and optical properties. Also, a novel seeding system, based on the atomization of a suspension of solid particles in the working fluid, was designed and implemented. The seeding system proved to be operational and suited for the purpose of accurately tracing non-ideal high temperature vapor flows. Nozzle flows of siloxane MM vapor were tested from non-ideal to almost ideal thermodynamic states, and measurements were taken at one single point along the expansion at three different conditions, corresponding to a subsonic ($M\approx 0.7$) non-accelerating flow, a supersonic ($M\approx 1.7$) non-accelerating flow, and a supersonic ($M\approx 1.4$) accelerating flow. The flow field was analyzed in terms of pressure ratio and velocity as a function of total conditions. Measured trends show a clear dependency on total conditions, thus proving the non-ideality of the analyzed flows. Also, experimental distributions of pressure ratio were compared to two-dimensional and viscous CFD simulations, while measured velocity was compared with both CFD results and values computed from pressure and temperature measurement using the isentropic expansion hypothesis.

Concerning measured pressure ratio, discrepancies from CFD are satisfactorily below 2.2% except for uniform flow at $M\approx 1.7$ where a difference of the order of 10% is observed, with a peak of 14.3% at the most non-ideal conditions. Such difference is probably to be partially ascribed to the influence of side wall boundary layer, which is not accounted for in the 2D simulations, and partially to a possible small fraction of kinetic head recovered due to tap edge undesired chamfering.

Regarding directly measured velocity, the observed discrepancy of CFD results is always below 3% except for the supersonic ($M\approx 1.4$) accelerating flow test, where the maximum deviation is 6.6% at the test start. The presence of impurities or agglomerated particles during the initial portion of the test could motivate an underestimation of measured velocity for the accelerating case; however, this hypothesis needs to be further investigated. Instead, the difference between CFD data and the velocity inferred from pressure and temperature measurements ranges between 4 and 5.4%, while discrepancy between LDV measured and velocity inferred from $P_T, T_T, P$ measurements is always below 4% except for the supersonic accelerating flow test.

The two different methods employed for measuring the velocity revealed to be congruent and also in accordance with the predictions of CFD simulations, thus proving the applicability of laser Doppler velocimetry for direct velocity measurement in high-temperature vapor flows with diverse levels of non-ideality, as well as the possibility of resorting to total and static pressure measurements to retrieve the flow velocity as it is required to develop directional pressure probes.
